# Prognostic Value of Normal Thyroid Stimulating Hormone in Long-Term Mortality in Patients With STEMI

**DOI:** 10.3389/fendo.2022.806997

**Published:** 2022-02-22

**Authors:** Lijie Sun, Keling Xiao, Zupei Miao, Yinghua Zhang, Jin Si, Ning Shi, Haoyu Zhang, Ting Zhao, Jing Li

**Affiliations:** ^1^Department of Geriatrics, Xuanwu Hospital Capital Medical University, National Clinical Research Center for Geriatric Diseases, Beijing, China; ^2^Department of Cardiology, Xuanwu Hospital Capital Medical University, Beijing, China

**Keywords:** thyroid stimulating hormone, ST-segment elevation myocardial infarction, mortality, prognosis, coronary artery disease

## Abstract

**Background:**

Although within the normal range, thyroid stimulating hormone (TSH) levels are associated with cardio-metabolic disorders and have an effect on the cardiovascular system. The aim of our study was to assess the prognostic value of normal TSH on long-term mortality in patients with ST-segment elevation myocardial infarction (STEMI).

**Methods:**

Consecutive STEMI patients who had a TSH level within the normal range (0.55–4.78 μIU/ml) were enrolled from November 2013 to December 2018. Patients were stratified into three groups depending on the tertile of TSH level, and all-cause mortality and cardiac death were compared. TSH concentrations associated with risk of all-cause mortality were evaluated in a continuous scale (restricted cubic splines) and the Cox proportional hazards regression model.

**Results:**

A total of 1,203 patients with STEMI were eligible for analysis. During a median follow-up of 39 months, patients in the 3rd tertile group had higher all-cause mortality (20.1% vs. 12.2% and 14.3%, p = 0.006) and cardiac death (15.4% vs. 7.7% and 12.3%, p = 0.001) as compared to the 1st and 2nd tertile groups. The Cox proportional hazards model showed that TSH was an independent predictor on long-term all-cause mortality (HR: 1.248, 95% CI: 1.046–1.490, p = 0.014). However, subgroup analysis indicated that TSH (HR: 1.313, 95% CI: 1.063–1.623, p = 0.012) was only significantly associated with long-term all-cause mortality in the patients without emergency reperfusion therapy. Restricted cubic spline analyses showed a linear relationship between TSH concentrations and all-cause mortality (P for non-linearity = 0.659).

**Conclusions:**

A Higher TSH level - even in a normal range is associated with long-term mortality in patients with STEMI, proposing an additional indication to identify STEMI patients with poor prognosis.

## Introduction

The mortality after ST-elevation myocardial infarction (STEMI) has dramatically decreased through reperfusion therapy and optimized antithrombotic treatment (1). However, it has always been a great challenge that STEMI patients are still at risk of major adverse cardiac events, even death after discharge. Therefore, current guidelines recommended some risk stratification models and biomarkers for identifying high-risk patients and more accurate secondary prevention (2–5).

Thyroid function exerts an important impact on the cardiovascular system. Overt and subclinical hyper- or hypothyroidism, manifested as low or high serum thyroid stimulating hormone (TSH) level, are associated with an increased risk of cardiovascular events and mortality (6–8). Interestingly, a previous study suggests that TSH levels in the upper part of the normal range are also positively associated with 30-day mortality in patients with coronary artery disease (CAD) after percutaneous coronary intervention (PCI) (9). So far, it is unclear whether TSH levels within the reference range have a predictive value on long-term prognosis in patients with STEMI. We therefore aimed to investigate the association of normal TSH levels with long-term mortality in patients following STEMI.

## Methods

### Study Population

**Figure 1:**
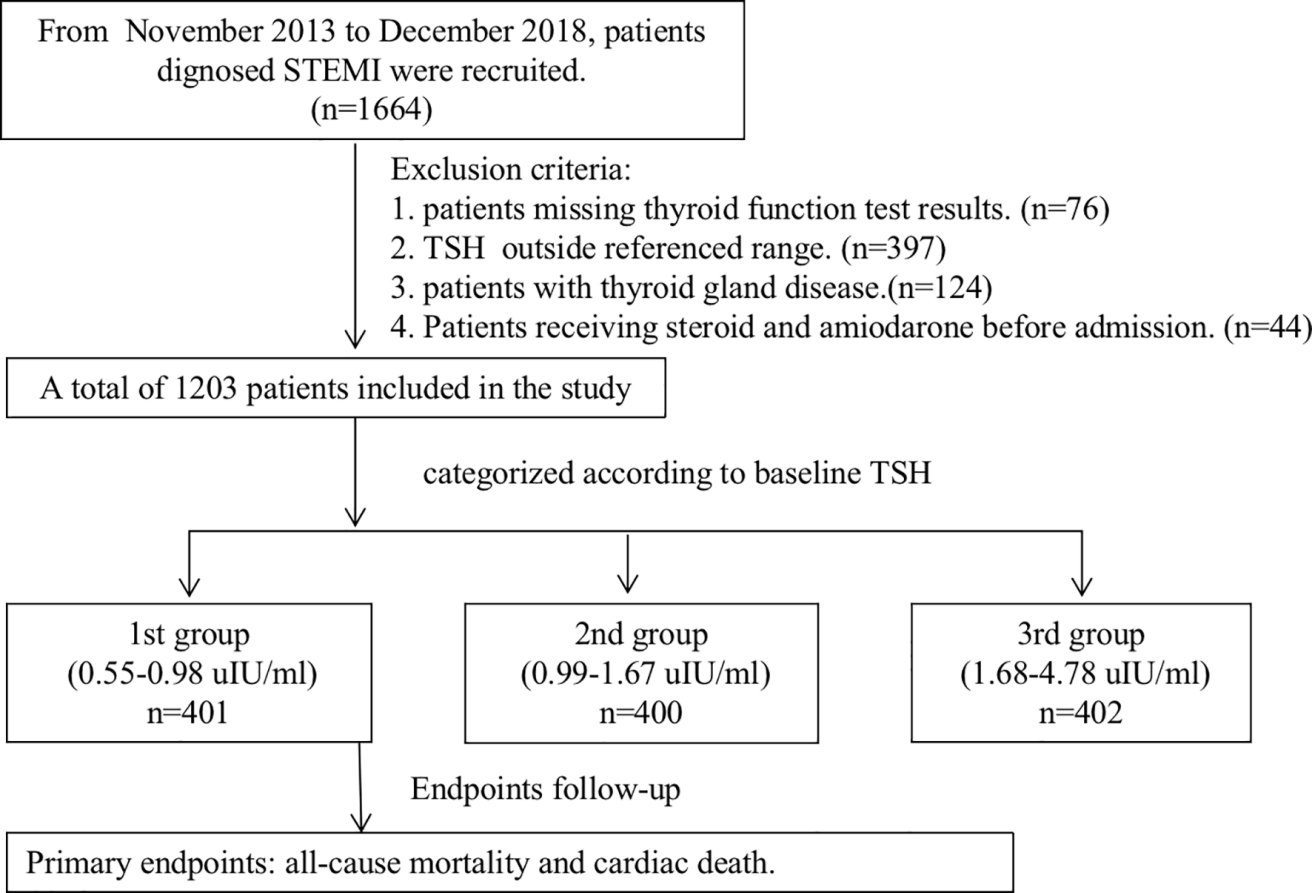
The study flowchart. STEMI, ST-segment elevation myocardial infarction; TSH, thyroid-stimulating hormone.

In this retrospective study, 1,664 consecutive patients hospitalized for STEMI were enrolled in a single center from November 2013 to December 2018. Only patients with a TSH level within the reference range (0.55 to 4.78 μIU/ml) were eligible for analysis. Patients were divided into three groups according to the tertile of the TSH level (**Figure 1**). Exclusion criteria consist of 1) missing thyroid function test results (n = 76); 2) prior or current thyroid disease (including prior history, surgery, or drug therapy for thyroid disease) (n = 124); 3) abnormal thyroid status and TSH beyond the reference range (n = 397); and 4) receiving steroid and amiodarone before admission (n = 44). Clinical information was collected from questionnaires and electronic medical records. Patients were treated according to standard clinical guidelines (10). Reperfusion therapy was indicated in patients with symptoms of ischemia of ≦12 h duration and persistent ST-segment elevation. A primary PCI strategy was recommended over fibrinolysis within indicated timeframes. However, if timely PCI cannot be performed after STEMI diagnosis, fibrinolytic therapy is recommended with 12 h of symptom onset in patients without contraindications. Our study complied with the Declaration of Helsinki, and written information consents were obtained from all participants. The study was approved by the ethics committee of Xuanwu Hospital Capital Medical University.

STEMI was defined as at least two of the three criteria: 1) presence of typical prolonged (≥20 min) chest pain; 2) significant ST-segment elevation (≧2 mm in at least 2 contiguous cordial leads, or ST-segment elevation ≧1 mm in at least 2 inferior leads) or new left bundle branch block; and 3) the elevation of myocardial biomarker (cTn or CK-MB) more than the 99th percentile or 2 folds of the normal value (11).

### Thyroid Function Testing

Blood samples were collected within 24 h of hospital admission. The thyroid function test includes serum TSH, total triiodothyronine (TT3), total thyroxine (TT4), free triiodothyronine (FT3), and free thyroxine (FT4) levels. After coagulation at room temperature for 30 min, samples were centrifuged at 4,000 rpm for 5 min and serum was aspirated. They were measured by chemiluminescent immunoassay on Centaur (Siemens, Erlangen, Germany).

### Follow-Up and Clinical Outcomes

Discharged patients were subsequently tracked *via* review of medical records and telephone interview at 6 and 12 months and then annually. The primary endpoint was all-cause mortality during follow-up. We further defined the death as cardiac and non-cardiac. Standard definitions were used for cardiac death (12).

### Statistical Analysis

Continuous variables were presented as mean (± SD) when normally distributed and median (25th–75th percentile) when not normally distributed. Categorical variables were shown as frequencies and percentages. The Shapiro–Wilk normality test was performed to test the normality of the data. Mean values of continuous variables were compared using one-way analysis of variance or Kruskal–Wallis tests, as appropriate. Proportions for categorical variables were compared by the chi-square test or Fisher exact test. Bonferroni correction was used to correct multiple comparisons. Univariable and forward stepwise multivariable Cox regression analyses were performed to clarify the independent prognostic impact of TSH on the all-cause mortality. The proportional hazards assumption was confirmed through Schoenfeld residuals (**Supplementary Files**). Baseline variables that were considered clinically relevant or that showed a univariate relationship with outcome were entered into the multivariate Cox proportional-hazards regression model. Variables for inclusion were carefully chosen, given the number of events available, to ensure parsimony of the final model. The association between TSH concentration in the reference range and all-cause mortality was evaluated on a continuous scale with restricted cubic spline curves (13) based on Cox proportional hazards models with 3 knots at the 10th, 50th, and 90th percentiles of TSH. HR was reported with a corresponding 95% CI. A two-sided p value <0.05 was considered to indicate statistical significance. Statistical analyses and charts were performed using the SPSS 25 (IBM SPSS Statistics 25, IBM Corporation, Armonk, NY, USA) and the R package.

## Results

### Baseline Data

A total of 1,203 consecutive patients were enrolled and divided into three groups according to the tertile of the TSH level, which were as follows: 1st group (TSH level from 0.55 to 0.98 μIU/ml, n = 401), 2nd group (TSH level from 0.99 to 1.67 μIU/ml, n = 400), and 3rd group (TSH level from 1.68 to 4.78 μIU/ml, n = 402). Baseline demographic and clinical characteristics are shown in **Table 1**. Age, male, high-risk Global Registry of Acute Coronary Events (GRACE) score, smoking history, the degree of pain relief, and the time of symptom onset to admission over 12 h were significantly different in patients of various groups. There was no difference in the occurrence of in-hospital mortality among three groups (2.5%, 4.5%, and 5.5%, p = 0.099).

**Table 1:** Baseline demographic and clinical characteristics.

Variables	1st group (n = 401)	2nd group (n = 400)	3rd group (n = 402)	p value
Age (years)	61 ± 13	62 ± 12	65 ± 13	**<0.001**
Male	320 (79.8%)	313 (78.3%)	283 (70.4%)	**0.004**
Smoking history	266 (66.3%)	247 (61.8%)	223 (55.5%)	**0.007**
Family history of CAD	113 (28.2%)	118 (29.6%)	82 (20.4%)	**0.006**
Hypertension	221 (55.1%)	242 (60.5%)	239 (59.6%)	0.253
Diabetes	123 (30.7%)	137 (34.3%)	140 (34.9%)	0.391
Hyperlipidemia	119 (29.9%)	132 (33.1%)	144 (36.1%)	0.178
SBP (mmHg)	132 ± 22	133 ± 22	133 ± 22	0.505
DBP (mmHg)	78 ± 15	79 ± 15	78 ± 15	0.789
Heart rate (/min)	76 ± 16	76 ± 17	77 ± 16	0.491
LVEF (%)	56.76 ± 9.87	57.57 ± 10.62	56.33 ± 10.03	0.236
GRACE score	147 ± 37	151 ± 83	157 ± 62	0.086
High risk group	197 (54.0%)	197 (52.3%)	232 (64.1%)	**0.002**
BMI (kg/m^2^)	26.37 ± 14.25	25.60 ± 3.53	28.05 ± 52.74	0.538
Killip class				0.401
I	226 (56.4%)	214 (53.5%)	214 (53.2%)	
II	140 (34.9%)	161 (40.3%)	159 (39.6%)	
III	23 (5.7%)	20 (5.0%)	23 (5.7%)	
IV	12 (3.0%)	5 (1.3%)	6 (1.5%)	
Degree of pain relief*^a^* (%)	60.14 ± 33.94	63.72 ± 33.03	66.90 ± 33.62	**0.026**
The time of symptom onset to admission over 12 h (%)	113 (29%)	138 (36.1%)	141 (37.8%)	**0.025**
Days in hospital	10 (8-13)	10 (8-13)	10 (8-14)	0.898
In-hospital mortality	10 (2.5%)	18 (4.5%)	22 (5.5%)	0.099

^a^Compared with the highest degree of pain.

Data are shown as mean ± standard deviation or n (%). Data in bold indicate p-values ≤ 0.05.

BMI, body mass index; DBP, diastolic blood pressure; SBP, systolic blood pressure; LVEF, left ventricular eject fraction; CAD, coronary artery disease; GRACE score, global registry of acute coronary events score.

Laboratory tests of three groups are shown in **Table 2**. The significant differences were found among three groups in terms of white blood cell counts (WBC), hemoglobin, creatine kinase isoenzymes (CK-MB), and D-dimer. Angiographic and procedural characteristics among three groups are shown in **Table 3**. Fewer patients in the 3rd group received reperfusion therapy compared with those in the other two groups (34.5% vs. 46.2% and 40.1%, p = 0.005). Numbers of lesion vessels and infarction-related artery were similar among three groups. In **Table 4**, no difference in medication prescription in hospital and after discharge was found in three groups.

**Table 2:** Laboratory variables at admission.

Variables	1st group (n = 401)	2nd group (n = 400)	3rd group (n = 402)	p value
WBC (10^9^/L)	10.56 ± 3.46	10.08 ± 3.11	9.40 ± 3.73	**<0.001**
Hemoglobin (g/L)	143 ± 21	142 ± 22	139 ± 19	**0.003**
Platelets (10^9^/L)	230.8 ± 69.1	226.7 ± 64.7	229.0 ± 67.2	0.692
D-Dimer (μg/ml)	0.31 (0.20–0.54)	0.31 (0.20–0.56)	0.36 (0.23–0.66)	**0.014**
CK-MB (IU/L)	87 (29–173)	49 (21–121)	31 (18–90)	**<0.001**
Creatinine (mg/dL)	72 (63–83)	73 (62–87)	71 (60–87)	0.275
Homocysteine (μmol/L)	14.8 (11.9–19.3)	15.1 (12.2–21.0)	14.9 (11.9–20.1)	0.951
LDL-cholesterol (mmol/L)	2.75 ± 0.84	2.77 ± 0.82	2.83 ± 0.93	0.440
Total cholesterol (mmol/L)	4.35 ± 1.01	4.35 ± 0.96	4.45 ± 1.10	0.350
HbA1c (%)	5.9 (5.5–6.9)	6.1 (5.6–7.5)	6.0 (5.6–7.1)	0.150
hsCRP (mg/L)	4.41 (1.74–13.61)	4.84 (1.54–14.87)	5.47 (2.18–15.4)	0.338
Interleukin-6 (pg/mL)	39.41 ± 56.27	37.54 ± 50.63	37.20 ± 59.44	0.836
Triiodothyronine (ng/mL)	0.88 ± 0.57	0.88 ± 0.52	0.89 ± 0.22	0.975
Thyroxine (μg/dL)	7.68 ± 1.88	7.68 ± 1.90	7.69 ± 2.05	0.997
Free triiodothyronine (ng/mL)	2.52 ± 0.48	2.54 ± 0.43	2.54 ± 0.46	0.253
Free thyroxine (μg/dL)	1.11 ± 0.20	1.12 ± 0.21	1.15 ± 0.67	0.252
TSH (μIU/mL)	0.76 (0.65–0.86)	1.27 (1.11–1.46)	2.44 (1.98–3.09)	**–**

Data are shown as mean ± standard deviation or n (%). Data in bold indicate p-values ≤ 0.05.

WBC, white blood cell; CK-MB, creatine kinase isoenzymes; LDH, lactate dehydrogenase; LDL-cholesterol, low-density lipoprotein-cholesterol; HbA1c, glycated hemoglobin; hsCRP, hypersensitive C-reactive protein; TSH, thyroid stimulating hormone.

**Table 3:** Angiographic and procedural characteristics.

Variables	1st group (n = 401)	2nd group (n = 400)	3rd group (n = 402)	p value
One-vessel disease	93 (23.2%)	94 (23.5%)	86 (21.4%)	0.918
Multivessel disease	245 (61.1%)	230 (57.5%)	220 (54.7%)	0.187
IRA				
LAD	127 (31.6%)	120 (30.0%)	108 (26.9%)	0.317
LCX	36 (9.0%)	26 (6.5%)	33 (8.2%)	0.413
RCA	84 (20.9%)	87 (21.8%)	81 (20.1%)	0.856
Emergent reperfusion therapy	175 (46.2%)	151 (40.1%)	129 (34.5%)	**0.005**
Number of stents implanted	2.2 ± 1.2	2.1 ± 0.8	2.2 ± 1.0	0.515

Data are shown as mean ± standard deviation or n (%). Data in bold indicate p-values ≤ 0.05.

IRA, infarction-related artery; LAD, left anterior descending; LCX, left circumflex; RCA, right coronary artery.

**Table 4:** Medications used in hospital and after discharge.

Variables	1st group (n = 401)	2nd group (n = 400)	3rd group (n = 402)	p value
Asprins	394 (98.3%)	395 (98.8%)	395 (98.3%)	0.811
Clopidogrel	379 (94.5%)	386 (96.5%)	380 (94.5%)	0.320
Ticagrelor	14 (3.5%)	12 (3.0%)	15 (3.7%)	0.844
Tirofiban	90 (22.4%)	89 (22.3%)	84 (20.9%)	0.846
Bivalirudin	21 (5.2%)	17 (4.3%)	17 (4.3%)	0.737
β-Blockers	336 (83.8%)	336 (84.0%)	329 (81.8%)	0.665
ACEI	351 (87.5%)	356 (89.0%)	351 (87.3)	0.728
ARB	23 (5.7%)	33 (8.3%)	30 (7.5%)	0.368
CCB	34 (8.5%)	52 (13.0%)	54 (13.4%)	0.053
Statins	393 (98.0%)	394 (98.5%)	395 (98.3%)	0.867
LMWH	394 (98.3%)	391 (97.8%)	390 (97.0%)	0.503
PPI	223 (55.6%)	209 (52.3%)	243 (60.4%)	0.063
Diuretics	185 (46.1%)	182 (45.5%)	184 (45.8%)	0.984
Antibiotic	79 (19.7%)	80 (20.0%)	92 (22.9%)	0.471
OAD	83 (20.7%)	106 (26.5%)	90 (22.4%)	0.135
Insulin	99 (24.7%)	105 (26.3%)	95 (23.6%)	0.689

Data are shown as n (%).

ACEI, angiotensin-converting enzyme inhibitors; ARB, angiotensin receptor blockers; CCB, calcium channel blockers; LMWH, low molecular weight heparins; PPI, proton pump inhibitors; OAD, oral antidiabetic drug.

### Clinical Outcomes

The median time of follow-up is 39 months, and the follow-up rate is 93.74%. The primary endpoint occurred in 187 patients: 49 (12.2%) patients in the 1st group, 57 (14.3%) patients in the 2nd group, and 81 (20.1%) patients in the 3rd group. Meanwhile, 62 (15.4%) patients in the 3rd group had higher cardiac death than 27 (6.7%) patients in the 1st group and 49 (12.3%) patients in the 2nd group (p < 0.001).

After adjusting the influence of other covariates incorporated in the multivariate Cox regression model (**Table 5**), we found that TSH was independently associated with the increased risk of all-cause mortality (HR: 1.248, 95% CI: 1.046–1.490, p = 0.014). Age, diabetes, Killip class, and hemoglobin retained statistical significance for all-cause mortality. All subjects were classified into two groups based on whether receiving emergency reperfusion therapy. After adjustment for confounding factors in a multivariate Cox regression analysis, hypertension (HR: 2.606, 95% CI: 1.225–5.541, p = 0.013) remained an independent predictor for patients with emergency reperfusion therapy, while age (HR: 5.360, 95% CI: 2.830–10.150, p < 0.001), diabetes (HR: 1.893, 95% CI: 1.219–2.938, p = 0.004), Killip class (HR: 2.760, 95% CI: 1.599–4.765, p < 0.001), and TSH level (HR: 1.313, 95% CI: 1.063–1.623, p = 0.012) retained statistical significance for patients without emergency reperfusion therapy (**Table 6**).

**Table 5:** Cox regression analysis on all-cause mortality in the long term.

Variables	Univariate analysis		Multivariate analysis	
	Unadjusted HR 95% CI	p value	Adjusted HR 95% CI	p value
Age	4.373 (2.837–6.740)	**<0.001**	3.632 (2.259–5.840)	**<0.001**
Male	1.799 (1.272–2.545)	**0.001**	0.948 (0.603–1.488)	0.816
Hypertension	1.427 (1.007–2.023)	**0.046**	1.336 (0.912–1.956)	0.137
Diabetes	1.811 (1.299–2.526)	**<0.001**	1.511 (1.048–2.178)	**0.027**
Smoking	1.658 (1.190–2.309)	**0.003**	0.895 (0.582–1.376)	0.613
Killip class	2.563 (1.750–3.755)	**<0.001**	2.956 (1.828–4.779)	**<0.001**
Emergency reperfusion therapy	0.697 (0.476–1.021)	0.064	0.824 (0.559–1.213)	0.326
TSH	1.271 (1.082–1.493)	**0.004**	1.248 (1.046–1.490)	**0.014**

Data in bold indicate p-values ≤0.05.

Adjusted for age (<60, ≥60), male, hypertension, diabetes, smoking, Killip class (I and II, III and IV), and emergency reperfusion therapy.

**Table 6:** Multivariate Cox regression analysis in the subgroup.

Variables	With emergency reperfusion therapy		Without emergency reperfusion therapy	
	Adjusted HR	p value	Adjusted HR	p value
	95% CI		95% CI	
Age	1.970 (0.945–4.107)	0.070	5.360 (2.830–10.150)	**<0.001**
Male	0.910 (0.418–1.984)	0.364	0.838 (0.473–1.486)	0.546
Hypertension	2.606 (1.225–5.541)	**0.013**	1.039 (0.663–1.626)	0.869
Diabetes	0.928 (0.474–1.818)	0.829	1.893 (1.219–2.938)	**0.004**
Smoking	0.506 (0.240–1.068)	0.074	1.153 (0.667–1.994)	0.610
Killip class	2.725 (0.953–7.791)	0.061	2.760 (1.599–4.765)	**<0.001**
TSH	1.177 (0.835–1.658)	0.352	1.313 (1.063–1.623)	**0.012**

Data in bold indicate p-values ≤0.05.

Adjusted for age (<60, ≥60), male, hypertension, diabetes, smoking, and Killip class (I and II, III and IV).

**Figure 2:**
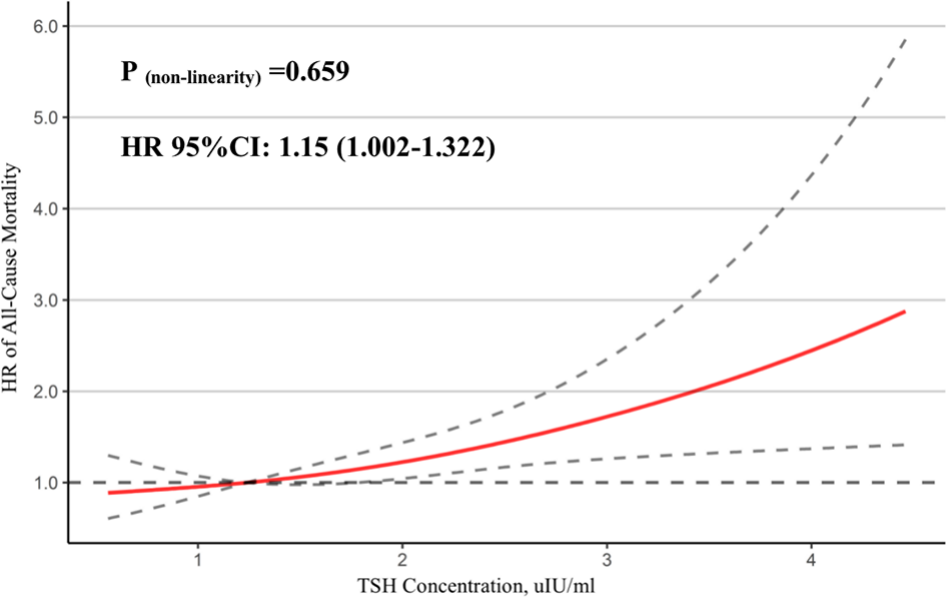
Association between TSH and mortality. A restricted cubic spline regression model was used to evaluate the relationship between normal TSH concentration and all-cause mortality in STEMI patients.

In **Figure 2**, we used a restricted cubic spline to flexibly model and visualize the relationship of TSH concentrations in the reference range with all-cause mortality in STEMI patients. Above 1.79 μIU/ml, TSH concentrations were positively associated with the risk of all-cause mortality (HR = 1.15, 95% CI: 1.002–1.322, P for non-linearity = 0.659).

## Discussion

The present study explored the relationship between TSH within the reference range and long-term mortality in patients with STEMI. The most important findings of the study can be summarized as the following: 1) in patients with STEMI, TSH in the highest tertile within the reference range was associated with a higher risk of long-term mortality, especially cardiac death, and 2) a high-normal TSH level at admission was an independent predictor of increased risk of mortality, especially in those without receiving reperfusion therapy.

The association between TSH level within the reference range and mortality has been reported in several longitudinal cohort studies. Ndrepepa et al. (9) enrolled 8,010 patients treated with PCI and found that the TSH level was associated with 30-day mortality but not with 30-day to 3-year mortality. Gürdoğan et al. (14) reported that the high-normal TSH group in euthyroid acute coronary syndrome (ACS) patients was an independent predictor for mortality during the 6-month follow-up. However, Åsvold et al. (15) analyzed 14 cohorts, including 55,412 patients with the TSH level within the reference range and no previously known thyroid or cardiovascular disease at baseline and concluded that TSH levels within the reference range were not associated with risk of cardiovascular events or CAD mortality. To our best knowledge, the current study is the first report demonstrating the relationship between normal TSH and long-term mortality in STEMI patients.

Taken together with our study and previous data, TSH may have a predictive value on patients with known CAD, who have more significant atherosclerosis and relatively higher risk of recurrent events than those without CAD. In our STEMI cohort, the impact of TSH changes was emphasized by disclosing a correlation of TSH with high-risk GRACE score, which represents an important clinical marker of worse prognosis in acute STEMI. Since in the 3rd TSH tertile group, there are more patients with time from symptom onset >12 h and fewer patients got primary PCI and fibrinolytic therapy, we conducted a subgroup analysis of reperfusion therapy. In subgroup analysis, the association between mortality and normal TSH level only persisted in STEMI patients without reperfusion therapy, who are at a higher risk of early complication and death. Consequently, TSH in the upper reference range can serve as an indicator for increased risk of long-term mortality in patients with STEMI.

The mechanism of association between TSH in the upper part of the reference range and increased risk of STEMI follow-up mortality is not clear. Some researchers reported that TSH levels in the upper part of the reference range are associated with endothelial dysfunction (16), unsteady systolic and diastolic blood pressure (17, 18), arterial stiffness (19), metabolic syndrome (20), and less favorable lipid levels (21). Meanwhile, studies have found that TSH can promote inflammation by stimulating adipose tissue pro-inflammatory cytokines (22), increase cardiometabolic disorders (23), affect heart remodeling after myocardial infarction (24), and increase higher thrombus burden (25). In addition, euthyroid sick syndrome, manifested normal TSH and decreased free T3 levels, are related to the increased mortality in both heart failure and acute myocardial infarction (26, 27). TSH in the upper part of the reference range may indicate a high-risk condition for STEMI patients, which should get more attention in clinical practice. In the current study, the contribution of these conditions in the relationship between normal TSH level and mortality remains unproven. Further investigation is needed to assess the mechanism of the normal TSH level in STEMI patients.

Delimitation of the reference range of TSH is controversial (28–30), and some people proposed to reduce the upper limit of reference to 2.5–3.0 mIU/l (31). Our study found that the TSH level was positively associated with the risk of all-cause mortality when above 1.79 μIU/ml. Inoue et al. (32) have reported that a U-curved association between TSH and the risk of all-cause mortality in the cohort study of 9,020 US adults and higher TSH levels (>1.96 mIU/l) were associated with increased risk of all-cause mortality. Gürdoğan et al. (14) reported that the high-normal TSH group (>1.60 μIU/ml) in ACS patients was an independent predictor for mortality during the 6-month follow-up. Evidence shows that although the serum TSH level reflects thyroid–pituitary feedback, it might not be efficient enough to show thyroid status in every organ (33). Based on this assumption, an appropriate pituitary FT4 level might not be suitable for the cardiovascular system and increases the incidence of CAD. Therefore, for CAD patients, the general reference range of TSH might not be reliable enough, and the continuous effect of TSH should be regarded as a risk factor. We look forward to more convincing studies to verify the relationship between TSH and mortality in STEMI patients, and to guide the division of TSH thresholds among different populations.

There are some limitations in our study. First, it is a retrospective and observational research with possible selection bias and confounding factors. Second, our TSH was measured by chemiluminescent immunoassay and the reference range is only response to this measurement. Moreover, TSH was monitored only once at admission; the relationship between TSH changes and mortality remained undefined without reexamination and regular monitoring. Thyroid function testing did not include thyroid autoantibodies, which was associated with clinical or subclinical thyroid diseases. Third, cardiac biomarkers in our study were not obtained at admission and thus might reflect the degree of cardiac injury insufficiently. Finally, the current study indicates that TSH is related to long-term mortality in STEMI patients, but the relevant mechanism was not explained thoroughly.

## Conclusion

TSH in the upper part of the reference range is associated with an increased risk of long-term mortality in STEMI patients, especially in those without reperfusion therapy.

## Data Availability Statement

The raw data supporting the conclusions of this article will be made available by the authors, without undue reservation.

## Ethics Statement

The studies involving human participants were reviewed and approved by the ethics committee of Xuanwu Hospital Capital Medical University. The patients/participants provided their written informed consent to participate in this study.

## Author Contributions

JL, LS, and KX contributed to the conception and design of the work. LS, XK, ZM, YZ, JS, NS, HZ and TZ contributed to the acquisition. JL, LS, ZM, HZ and KX drafted the manuscript. All authors contributed to the article and approved the submitted version.

## Funding

The work was funded by the National Natural Science Foundations of China (grant number 81470491 and 82170347) and the Beijing Municipal Natural Science Foundation (grant number 7192078).

## Conflict of Interest

The authors declare that the research was conducted in the absence of any commercial or financial relationships that could be construed as a potential conflict of interest.

## Publisher’s Note

All claims expressed in this article are solely those of the authors and do not necessarily represent those of their affiliated organizations, or those of the publisher, the editors and the reviewers. Any product that may be evaluated in this article, or claim that may be made by its manufacturer, is not guaranteed or endorsed by the publisher.

## Abbreviations

STEMI, ST-elevation myocardial infarction; TSH, thyroid stimulating hormone; CAD, coronary artery disease; PCI, percutaneous coronary intervention; TT3, total triiodothyronine; TT4, total thyroxine; FT3, free triiodothyronine; FT4, free thyroxine; GRACE, Global Registry of Acute Coronary Events; WBC, blood cell counts; CK-MB, creatine kinase isoenzymes; LDL, low-density lipoprotein; ACS, acute coronary syndrome.
