# Supplementary material for: Prognostic Value of Normal Thyroid Stimulating Hormone in Long-Term Mortality in Patients With STEMI
Source: Front Endocrinol (Lausanne). 2022 Feb 22;13:806997. doi: 10.3389/fendo.2022.806997 (PMC8902238; doi:10.3389/fendo.2022.806997)
Supplement: Supplementary file 1 [file DataSheet_1.docx]

**Supplement data**

1. Schoenfeld residuals for continuous variables

| Variables | r* | P values |
| --- | --- | --- |
| TSH | -0.046 | 0.592 |
| Age | 0.056 | 0.513 |
| CK-MB | 0.030 | 0.730 |
| D-dimer | -0.079 | 0.357 |
| Hemoglobin | 0.068 | 0.423 |

*Pearson correlation was used for linear correlation analysis between partial residual and rank of time.

1. Kaplan-Meier method for categorical variables


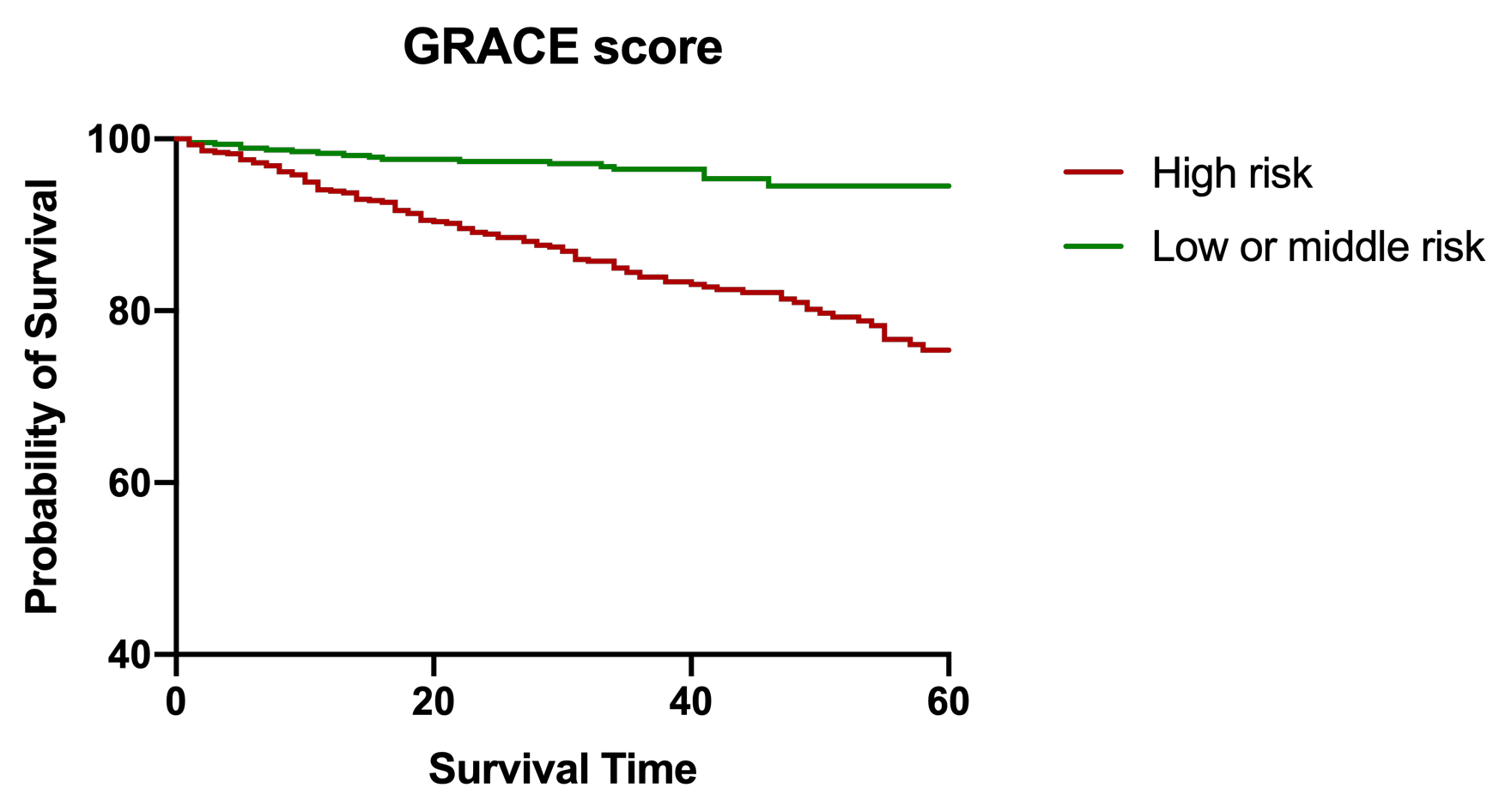

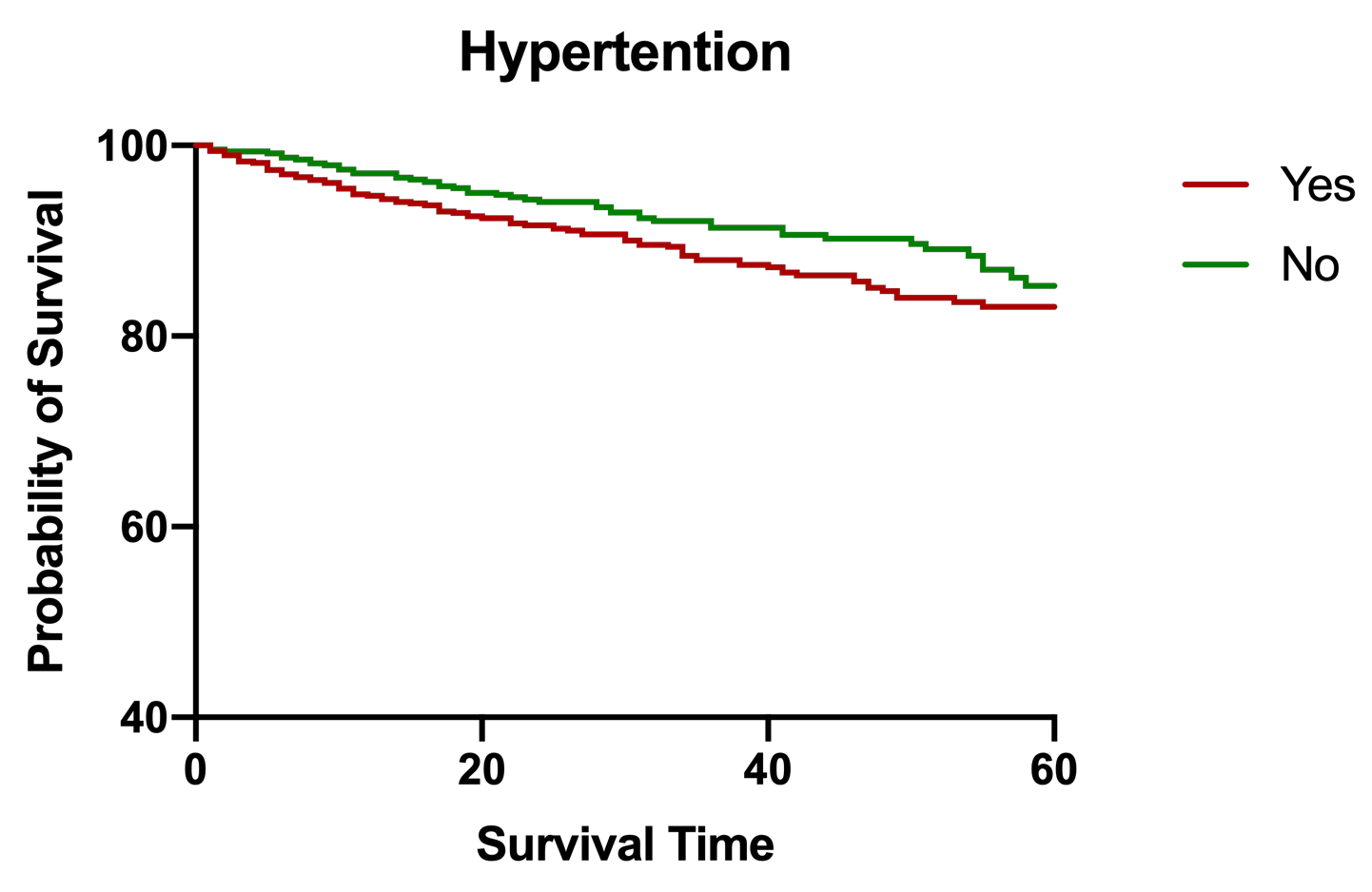

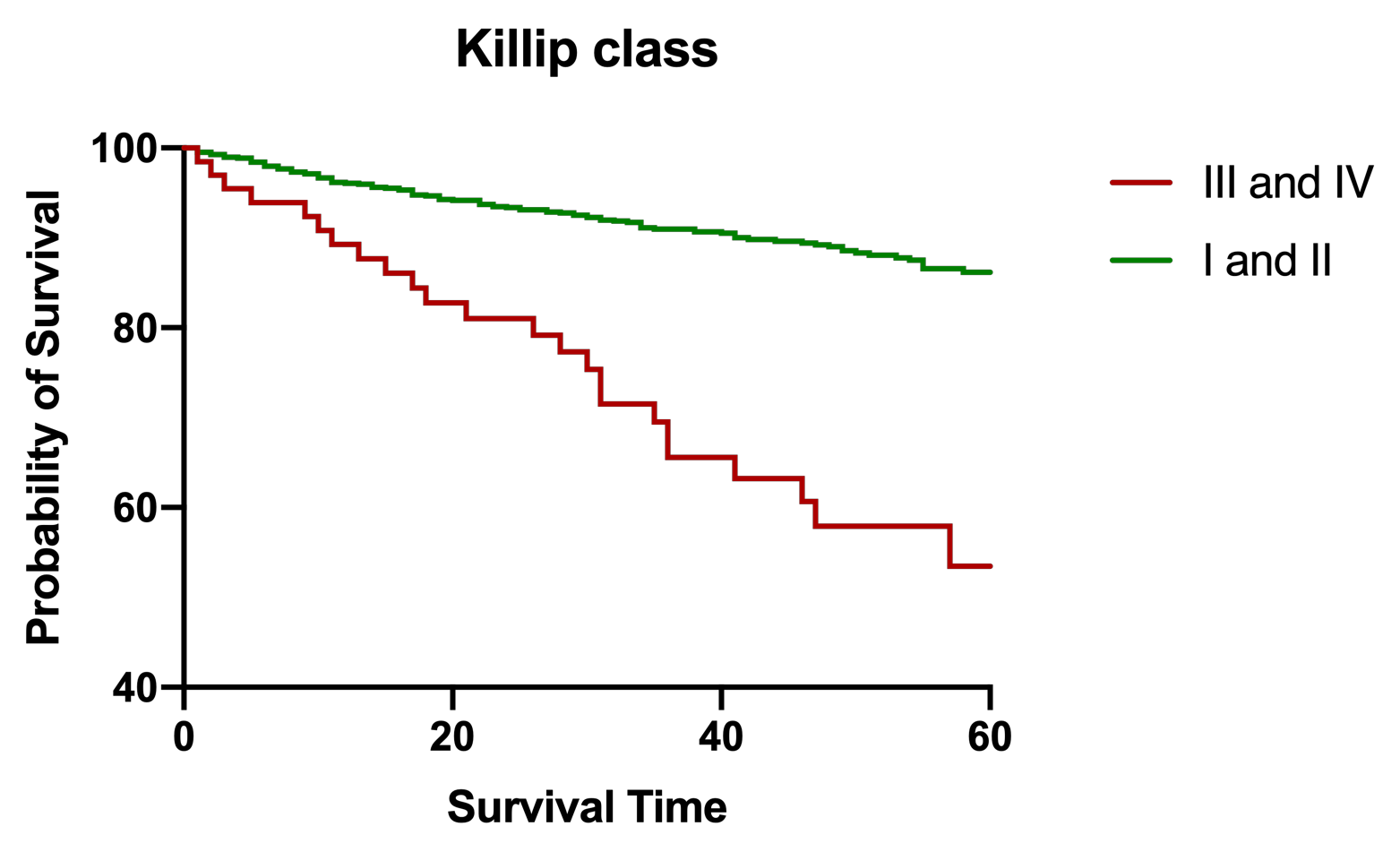

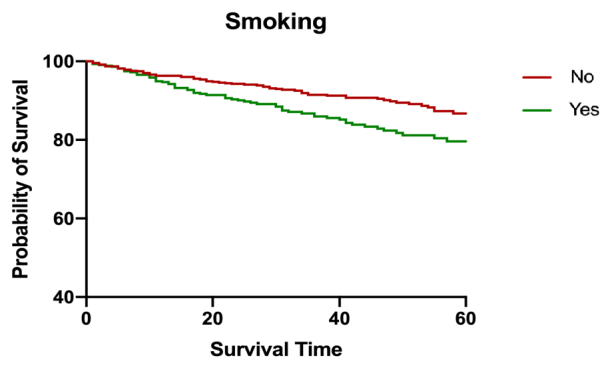

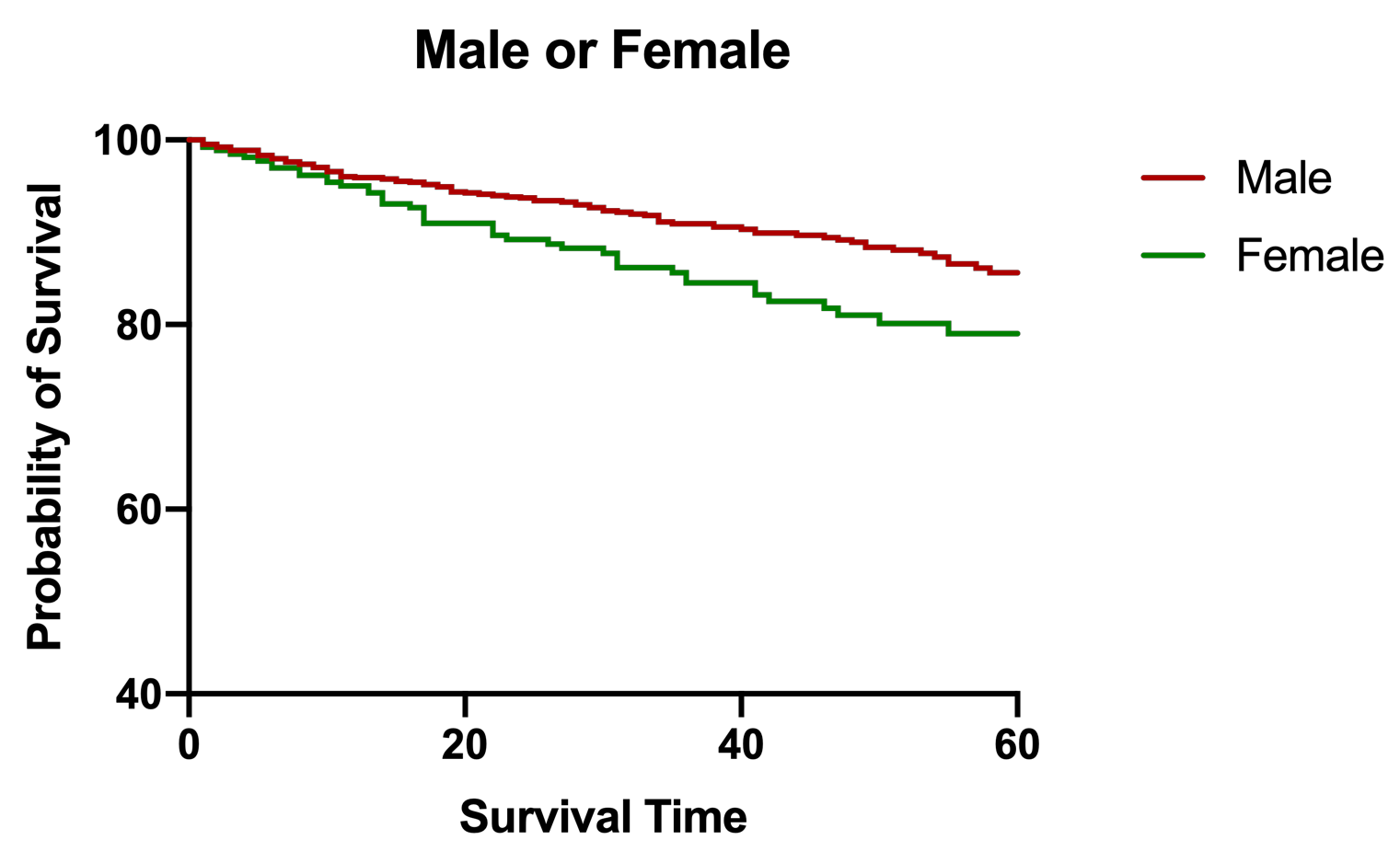


| Time-dependent Covariables | HR  95% CI | P values |
| --- | --- | --- |
| LN(T_)*TSH | 1.037(0.88-1.222) | 0.664 |
| LN(T_)*Age | 1.001(0.985-1.016) | 0.942 |
| LN(T_)*Male | 1.023(0.716-1.461) | 0.901 |
| LN(T_)*Hypertension | 1.269(0.875-1.839) | 0.209 |
| LN(T_)*Diabetes | 1.027(0.735-1.435) | 0.877 |
| LN(T_)*Smoking | 1.095(0.784-1.531) | 0.593 |
| LN(T_)*Killip class | 0.836(0.531-1.315) | 0.438 |
| LN(T_)*GRACE high-risk | 0.917(0.577-1.460) | 0.716 |
| LN(T_)*CK-MB | 1.001(0.999-1.002) | 0.659 |
| LN(T_)*D-dimer | 0.991(0.949-1.035) | 0.696 |
| LN(T_)*Hemoglobin | 1.000(0.995-1.006) | 0.855 |

1. Time-dependent Cox regression model to verify every variable
